# Bean common bacterial blight: pathogen epiphytic life and effect of irrigation practices

**DOI:** 10.1186/2193-1801-2-41

**Published:** 2013-02-08

**Authors:** Alireza Akhavan, Masoud Bahar, Homa Askarian, Mohammad Reza Lak, Abolfazl Nazemi, Zahra Zamani

**Affiliations:** 1 Dept. of Plant Protection, College of Agriculture, Isfahan University of Technology, Isfahan, Iran; 2 Plant Pests and Diseases Res. Division, Agricultural and Natural Resources Res. Center, Arak, Iran; 3 Dept. of Agricultural, Food and Nutritional Science, University of Alberta, Alberta, Canada

**Keywords:** Bean common Bacterial blight, Epiphytic life, Irrigation, Disease severity, *Xanthomonas axonopodis* pv. *phaseoli*

## Abstract

In recent years, bean common bacterial blight (CBB) caused by *Xanthomonas axonopodis* pv. *phaseoli* (*Xap*) has caused serious yield losses in several countries. CBB is considered mainly a foliar disease in which symptoms initially appear as small water-soaked spots that then enlarge and become necrotic and usually bordered by a chlorotic zone. *Xap* epiphytic population community has a critical role in the development of the disease and subsequent epidemics. The epiphytic population of *Xap* in the field has two major parts; solitary cells (potentially planktonic) and biofilms which are sources for providing and refreshing the solitary cell components. Irrigation type has a significant effect on epiphytic population of *Xap*. The mean epiphytic population size in the field with an overhead sprinkler irrigation system is significantly higher than populations under furrow irrigation. A significant positive correlation between the epiphytic population size of *Xap* and disease severity has been reported in both the overhead irrigated (r=0.64) and the furrow irrigated (r= 0.44) fields.

## Introduction

Pulse legumes are a very critical protein source in many developing countries. Among them, common bean (*Phaseolus vulgaris* L.) is consumed worldwide as a main source of protein, particularly in most Latin-American and African countries (Reynoso-Camacho et al. 2006).

**Figure 1:**
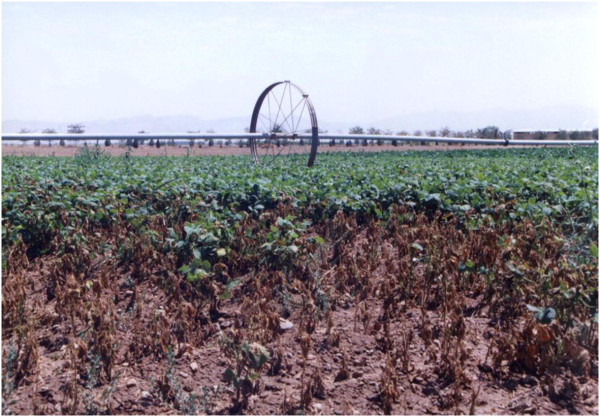
**High disease severity showing the consequences of bacterial dispersal by overhead sprinkler irrigation, central part of Iran.**

Several bacterial diseases infect common bean including common bacterial blight (CBB), halo blight, and bacterial brown spot caused by *Xanthomonas axonopodis* pv. *phaseoli* (*Xap*), *Pseudomonas syringae* pv. *phaseolicola* and *Pseudomonas syringae* pv. *syringae*, respectively. Although all three are destructive and economically important, CBB seems to be more widespread and causes relatively more yield loss (Hall 1994). This disease can cause up to 40% yield loss (Opio et al. 1996) and is still considered a major constraint to dry bean production in many countries and in particular Argentina, Brazil, Columbia, Mexico, Uganda, Zambia, Zimbabwe, South Africa, United States and recently Iran (Gilbertson and Maxwell 1992; Fourie 2002; Lak et al. 2002; Harveson 2009; Zamani et al. 2011; Karavina et al. 2011). In Iran, CBB now is one of the major bean diseases in three provinces in the central part of the country (Zamani et al. 2011). It was initially reported in 2002 from bean farms with furrow irrigation system, but as a rare non-destructive disease in Markazi province in the central part of the country where 17339 hectares of pintos and kidneys bean fields were located (Lak et al. 2002). In following years, due to lack of sufficient sources of water in the province and overlooking *Xap* existence in the area, farmers have been encouraged to stop applying furrow irrigation and to employ overhead sprinkler irrigation due to its higher efficacy in term of water use. Overhead irrigation was widely accepted by farmers and many bean growing fields replaced furrow irrigation with overhead sprinkler. Up to 2007, large epidemics of the disease have frequently occurred in the province of Markazi leading to huge yield losses (Osdaghi et al. 2010), and typically in large fields equipped with overhead sprinkler irrigation (Figure 1).

### Common bacterial blight symptoms

**Figure 2:**
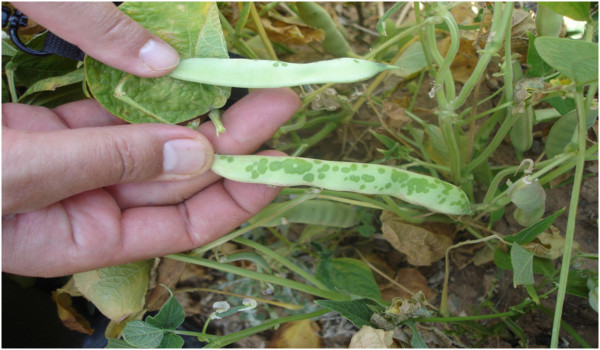
**Initial water soaked spots on bean pods.**

**Figure 3:**
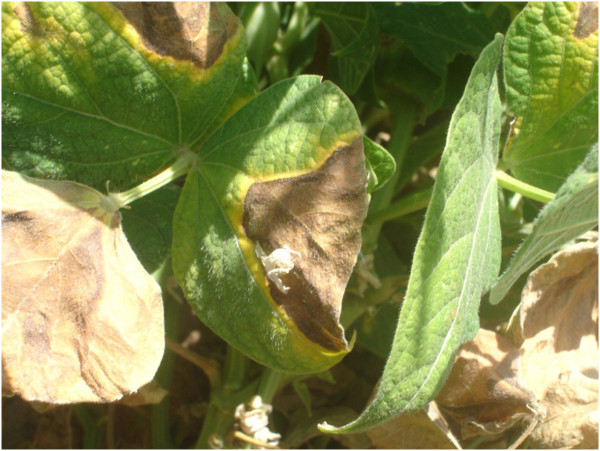
**Enlarged necrotic lesions on bean leaves bordered by a chlorotic zone.**

**Figure 4:**
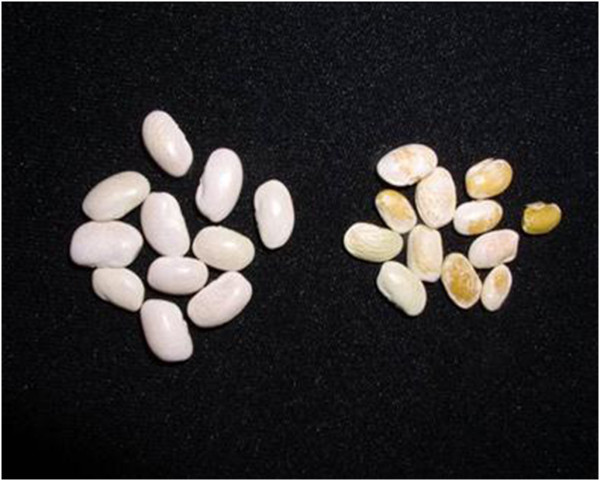
**Infected symptomless (left) and symptomatic seeds (right).**

Although CBB is considered mainly a foliar disease, symptoms can also be observed on stems, pods and seeds, symptoms initially appear as small water-soaked spots (Figure 2), which then enlarge and become necrotic and are usually bordered by a yellow zone in case of leaf spots (Figure 3) (Gilbertson and Maxwell 1992; Hall 1994; Harveson 2009). Bacterial ooze exuding from infected bean leaves can easily be observed using a compound microscope. Individual lesions may grow together causing plants to look burned, spots on pods are usually circular and brownish red, while infected seeds develop yellow to brown spots and show weak vigour and germination (Gilbertson and Maxwell 1992). Sometimes seeds have no visible symptoms and can germinate vigorously while they support large symptomless epiphytic communities of the pathogen (Figure 4) (Akhavan et al. 2009a).

### The causal agent of common bacterial blight

**Figure 5:**
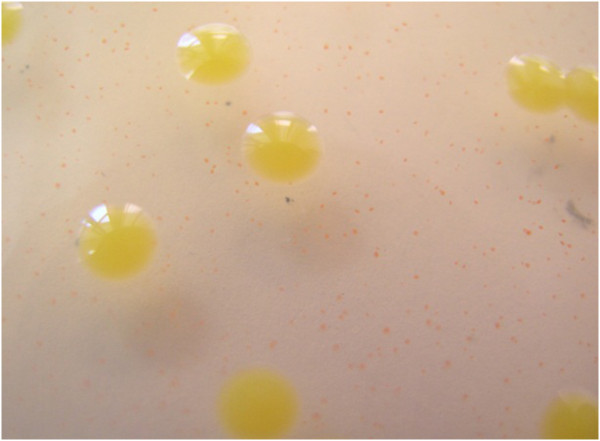
**Bacterial colonies of*Xap*on Modified NBY; a new developed semi selective medium.**

CBB is caused by *Xanthomonas axonopodis* pv. *phaseoli* (also known as *Xanthomonas campestris* pv*. phaseoli*) and it’s variant; *Xanthomonas axonopodis* pv. *phaseoli* var. *fuscans* about both, comprehensive taxonomical information have been published in many studies including Vauterin et al. (1995), Schaad et al. (2000), Vauterin et al. (2000) and Schaad et al. (2005). These two variants are identical in term of their epidemics and disease cycle but colonies of *X. axonopodis* pv. *phaseoli* var. *fuscans* are distinguished by a distinct brown pigmentation in media containing tyrosine, 2 to 9 days after inoculation (Goodwin and Sopher 1994). Typically, 3–5 days old colonies of *Xap* on regular media e.g. NBY (Nutrient broth 8gr, Yeast extract 0.7gr, KH2PO4 2gr, K2HPO4 0.5gr, Glucose 1gr, 1 M MgSO4 1 ml, Agar 20gr) are convex, yellow and transparent (Figure 5), while individual cells are motile, aerobic, gram-negative, and rod-shaped with a single polar flagellum (Vidaver 1967; Schaad 2001).

### Plant penetration

Cells of *Xap* can enter bean plants through openings such as stomata in leaves and other plant organs and through hydathodes at leaf margins, wounding of plants, such as that created by wind-blown soil particles can create pores for bacteria entry (Rudolph 1993). Bacterial cells are also readily transmitted mechanically, especially when plants are wet, while arthropods may transmit the bacterium from plant to plant (Kaiser and Vakili 1978; Lindemann and Upper 1985). The bean stem can also be penetrated in three ways: i.e., via the stomata, vascular system of the leaf and from infected cotyledons (Kaiser and Vakili 1978). Bacterial cells can also enter seeds via the vascular system or through the pedicel, while infection of the young plant occurs when internally infected seed germinates and the bacterium is transmitted from the seed to seedling (Gilbertson and Maxwell 1992; Saettler 1989a).

### Favourable environmental conditions

In general, *Xap* causes very severe disease under high rainfall and humidity and warm temperature conditions (25-35°C) with maximum development occurring around 28°C (Gilbertson and Maxwell 1992; Saettler 1989a). Dissemination in the field is facilitated by wind-driven rain, while insects, people and contaminated equipment can be considered vectors (Gilbertson and Maxwell 1992; Saettler 1989a). Overhead sprinkler irrigation like high rainfall may provide a mean for bacterial dispersal, unlike furrow irrigation (EPPO/CABI 1996; Harveson 2009). Splashing water spreads the bacterial pathogen from diseased plants to healthy plants (Lindemann and Upper 1985). However, increases in relative humidity may not facilitate CBB epidemics. For example, it has been shown that a 20 percent difference in relative humidity (53% vs. 73%) did not significantly affect the *Xap* epiphytic population size and number of bacterial spots per plant in the greenhouse under controlled conditions (Akhavan et al. 2009a).

### Primary inoculum

**Figure 6:**
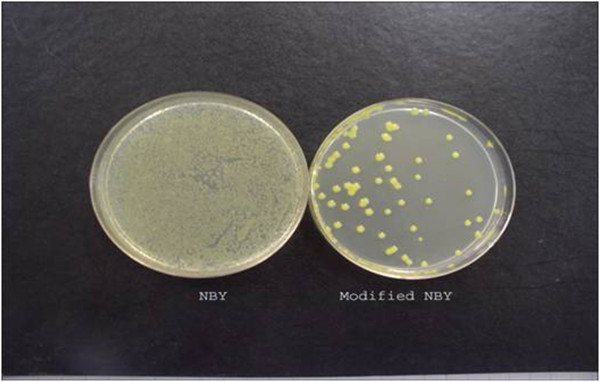
**Comparison of NBY and Modified NBY efficiency in isolation of*Xanthomonas axonopodis*pv*phaseoli*from symptomless bean leaves when leaves were washed and the same dilution cultured on both media at the same time.** Saprophytes grew on common NBY while, failed to grow on Modified NBY.

CBB is carried both on (externally) and in (internally) seed, in bean crop debris, and epiphytically on volunteer beans and perennial alternate host plants (Gilbertson and Maxwell 1992). Among all means, contaminated seed is probably the major source of bacteria introduced into new bean fields (Gilbertson and Maxwell 1992; Saettler 1989a). *Xap* remains viable for years under the seed coat (Saettler 1989a; EPPO/CABI 1996). Infected seed from a single crop may contaminate a significant area when used as a seed source; where one diseased plant in 10,000 is sufficient to cause a severe epidemic (EPPO/CABI 1996). Therefore, using pathogen-free seed is an important factor in disease management. The capability of several detection methods including Indirect ELISA, Direct PCR, Bio-PCR and Ic-PCR were compared for monitoring *Xap* in bean seeds (Akhavan et al. 2009b). The results indicated that sensitivity of Indirect ELISA was low and at least 10^5^ colony-forming unit/ml (cfu/ml) were needed as a detection threshold. In this study; the results for direct PCR were not necessarily reproducible, while Ic-PCR was found to be an expensive method. The Bio-PCR technique was considered as a reliable and specific method which was able to detect as little as 1 cfu/ml in seed extracts plated on a semi-selective medium called modified NBY (Figure 6) (Nutrient broth 8 gr, Yeast extract 0.7 gr, KH2PO4 2 gr, K2HPO4 0.5 gr, Glucose 1 gr, 1 M MgSO4 1 ml, Agar 20 gr, Cephalexin 25 mgr, 5-Fluorouracil 6 mgr, Cycloheximide 75 mgr and Nitrofurantoin 2mgr) (Vidaver 1967; Akhavan et al. 2009b). It has also been confirmed later by other researchers that the Bio-PCR assay is suitable for sensitive and routine testing of seed samples of beans for the presence of *Xap* (Osdaghi et al. 2010).

Survival of the pathogen in soil or plant debris is influenced by geographical area, climate, cultural practices, host genotypes, and bacterial strains (Karavina et al. 2008). *Xap* may survive in crop debris in the soil from season to season (Arnaud-Santana and Pena-Matos 1991; Gilbertson et al. 1990). However, such survival might not be realistic in most major bean growing areas of the world where the non-cropping period occurs under conditions where decomposition of crop debris is rapid and almost complete. Significant populations of *Xap* would not be expected to survive beyond six weeks under such conditions (Pernezny and Jones 2002) while in Zimbabwe, it was shown that *Xap* can over-winter between crops in crop residues; therefore, residues can be considered as sources of inocula for CBB in that country (Karavina et al. 2008). *Xap* can also survive and multiply as an epiphyte or resident on the shoot surfaces of weed hosts, primarily members of the legume family without showing symptoms (Pernezny and Jones 2002). In the Dominican Republic, *Xap* has been detected on *Euphorbia heterophylla* (L.), *Acanthospermum hispidum* (D.C.) and *Portulaca oleracea* (L.) (Angeles-Ramos et al. 1991). In Tanzania and Uganda, Saettler reported the pathogen on *Chenopodium album* (L.), *Solanum nigrum* (L.), *Echinochloa crus-galli* (L.), *Beta vulgaris* (L.) and *Amaranthus retroflexus* (L.) (Saettler 1989b).

### The secret life of *Xanthomonas axonopodis* pv. *phaseoli* as a foliar bacterial pathogen on leaves (symptomless colonization of leaves and biofilm formation)

A large number of foliar bacterial pathogens are able to survive and multiply on aerial parts of plants without any visible symptoms (Andrews and Harris 2000). It was demonstrated previously that *Xap* could survive both epiphytically and endophytically (Weller and Saettler 1980). The epiphytic population community has a very critical major role in the development of the disease and subsequent epidemics (Beattie and Lindow 1999). This symptomless period can lead to such a huge bacterial population that disease can develop later when more favorable environmental conditions occur (Wilson et al. 1999).

*Xap* is a seed-borne pathogen with an epiphytic symptomless population and is able to go through a long epiphytic phase on bean plants (Beattie and Lindow 1995). In general, the epiphytic population of *Xap* in the field has two major parts; solitary cells (potentially planktonic) and biofilms. Using ERIC fingerprinting, it has been shown for *Xap* that strains in the two fractions of the population are genetically identical (Jacques et al. 2005). A similar result has been demonstrated for strains of plant-associated *Pseudomonas fluorescens* (Boureau et al. 2004). Biofilms which are present on the surfaces of leaves are similar to those in aquatic ecosystems and hospital environments and they include a large aggregation of bacterial cells embedded in polymeric materials like extracellular polysaccharides. Comprehensive reviews on biofilm formation by plant-associated bacteria were published by Danhorn and Fuqua (2007) and Morris and Monier (2003). Among the bacteria which can form biofilms, one which is closest to *Xap*, is *X. campestris* pv. *campestris* in which there is a cell to cell signalling procedure. This system is coded by rpf genes cluster, which were previously known as an important cluster in the pathogenicity of bacterium (Crossman and Dow 2004). These genes have a role in production of diffusible factors like butyrolactones. The most well-known chemical in biofilm formation in many bacteria is called Acyl Homoserine Lactones (AHL), but it has not been found in the genus *Xanthomonas*; even though this molecule has an important role in biofilm formation of another bean bacterial pathogen; *Pseudomonas syringae* pv*. syringae* which has the same ecological cycle as the causal agent of CBB (Cha et al. 1998; Crossman and Dow 2004; Dow et al. 2003). Instead of AHL, butyrolactones may act as signal molecules in quorum-sensing-like systems in *Xanthomonas* (von Bodman et al. 2003; Jacques et al. 2005). Regarding *Xap,* AHL has not been found in the bacterium population; however, butyrolactones have been confirmed to have a role in biofilm formation (Jacques et al. 2005)*.*

### Cell viability within biofilms and its role in providing inoculum

The microbial epiphytic community of *Xap* needs to reach a threshold to be able to enter the leaves through natural openings like stomata or wounds and establish an endophytic population which leads to development of the disease (Beattie and Lindow 1999). In Michigan, this threshold has been indicated to be 2.5*10^5^ cfu per centimetre of bean leaves for *Xap* (Weller and Saettler 1980). It has been shown that the biofilm component looks stable following an initial period of growth of the *Xap* microbial community with population estimates of around 10^5^ cfu per gram of bean leaves, which is likely under the population threshold needed for disease development (Jacques et al. 2005). In contrast, it seems that solitary cell components of the population are responsible for plant infection and these biofilms are a reliable source to support the development of solitary cells. Biofilms are not easily influenced by any antimicrobial factors while solitary cells can be harmed by any antimicrobial environmental factors. It is now clear that populations of solitary cells under unfavorable conditions are easily influenced by a number of abiotic and biotic factors (Monier and Lindow 2003). In general, solitary cells are sensitive to a series of environmental factors such as temperature and UV radiation and also antimicrobial chemicals. Bacteria harbored in biofilms can easily resist any copper based chemicals since the extracellular polysaccharides of the biofilm can bind the chemicals while most solitary cells are sensitive to these compounds (Costerton et al. 1995). In the same way, antibiotics may not be effective tools against biofilms. Regarding UV radiation of different wavelengths, bacteria in biofilms can be physically protected since the polysaccharides intercept the radiation and thus the embedded cells are not exposed to UV radiation (Davey and O’ Toole 2000). In the case of *Xap,* it has been demonstrated that desiccation stress had no significant effect on biofilm population size, while solitary cell populations are drastically decreased by desiccation, overall, aggregation of bacterial cells in biofilms can protect them against unfavourable environmental conditions (Jacques et al. 2005; Amano 2010) while the same conditions can be lethal for solitary cells. A similar trend has been demonstrated for another destructive bean bacterium; *P. syringae* pv. *syringae* (Monier and Lindow 2003)*.* Cells of *Xap* aggregated in biofilms constitute a more stable population than do solitary cell populations. In *Xap*, biofilm population sizes are always lower than solitary population sizes; in contrast, it was shown that solitary cell populations which provide the bacteria that enter the plant through potential pores can multiply sharply when favorable conditions occur and even right after unfavorable circumstances (Jacques et al. 2005). This scenario raises the hypothesis that biofilms are sources for providing and refreshing the solitary cell components of epiphytic communities of plant pathogenic bacteria (Boureau et al. 2004; Jacques et al. 2005). For instance, it has been demonstrated that a reduction in hydric stress, i.e. excessive moisture, allowed solitary bacterial populations to increase again and it was suggested that biofilms were reservoirs for establishing solitary cell populations (Jacques et al. 2005).

### The effect of irrigation system on epiphytic population size and disease severity

In the field, rainstorms can usually be related to rises in bacterial population sizes to the threshold level with subsequent rapid disease development. The effect of rainstorms on *Xap* epidemics could be result of a sudden decrease of temperature or a rapid inflow of water or raindrop occurrence (Hirano et al. 1996; Jacques et al. 2005). It has been shown that epiphytic population of *Xanthomonas campestris* pv. *vesicatoria*, the cause of pepper bacterial leaf spot increased drastically following a 2-day wind-driven rain (Bernal and Berger 1996). Overhead sprinkler irrigation seems to have similar effects on bacterial population sizes as rainstorms. It has been demonstrated that the type of irrigation had a significant effect on epiphytic populations of *Xap*. Although the bacterial populations were the same size at the beginning, the mean epiphytic population size in the field with an overhead sprinkler irrigation system was 1.04*10^6^ colony forming unit per each squared centimeter of bean leaves (cfu/cm2) while for a furrow irrigation system, population size was 4.89*10^4^ cfu/cm^2^ (Akhavan et al. 2009a).

**Figure 7:**
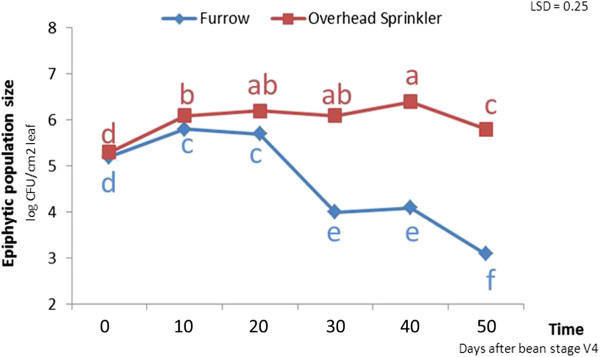
**In furrow irrigation,*Xap*population size (log cfu/cm2 leaf) increased up to just 10 days after the third trifoliate leaf unfolded (V4) following an initial inoculation at the second trifoliate stage while for overhead sprinkler irrigation, the population size increased up to 40 days.** Means with different letters are significantly different. The experiments were conducted in two different fields in the same research farm using a randomized complete block design with eight replicates in Arak, Iran in 2005.

It seems that overhead irrigation systems favour pathogen dissemination from colonized to healthy leaves as the key factor in increasing the total field epiphytic population size. In terms of multiplication, overhead irrigation provides a layer of water on the host plant surface, which results in an ideal multiplication site for the microbial planktonic community on plant leaves. Studies of other foliar bacterial pathogens have also confirmed that epiphytic population size can increase when the plant surfaces are wet (Beattie and Lindow 1995). The presence of a film of water also helps to promote nutrient release from plant leaves, which can enhance the availability of nutrient sources for the epiphytic bacterial community (Hirano and Upper 1983). Plant age by itself was found to have no effect on epiphytic *Xap* populations (Karavina et al. 2011). For *Xap*, it was indicated in another study that 10 percent of the total epiphytic population size particularly the solitary cells can move easily by rain (Mabagala 1997). In general, we can conclude that sprinkler irrigation has effects similar to rain; it can lower leaf and canopy temperature, increase relative humidity, and may prolong dew periods; it can enhance splash dispersal drastically; and it may remove cells from the atmosphere and deposit them on susceptible plant surfaces. However, one also has to notice the association of favourable weather conditions and the frequency of irrigation. The use of furrow rather than overhead irrigation systems in low rainfall areas has been found to restrict the spread of splash-dispersed pathogens (Baker 1980), and furrow irrigation is recommended in these areas to restrict pathogen spread (Icishahayo et al. 2007). It has been demonstrated that with furrow irrigation, the population of *Xap* on bean leaves only increased up to just 10 days after the third trifoliate leaf unfolded (V4) (Fernandez et al. 1986) following an initial inoculation at the second trifoliate stage leading to the appearance of symptoms on a few bean leaves. Infected bean leaves then senesced and even fell off of the plants before the “beginning bloom stage”, while the remaining plant leaves looked almost normal. In contrast, with overhead sprinkler irrigation; *Xap* populations increased up to 40 days, which coincided with the “pod filling stage” (Figure 7), while eventually destroying almost the whole bean crop (Akhavan et al. 2009a).

It has been revealed that irrigation type significantly influenced disease severity. Disease severity means were 5.8 and 2.8 in fields with overhead sprinkler irrigation and furrow irrigation, respectively using the standard system for the evaluation of bean germplasm with 1 as “no visible disease symptoms” and 9 as “very severe disease symptoms”(Schoonhoven and Pastor-Corrales 1994; Akhavan et al. 2009a). In addition to dispersing bacterial cells and helping them to reach healthy plants as the key factor, and promoting release of leaf nutrients to the microbial community, overhead sprinkler irrigation can generate a film of water over the leaf surface including stomata, providing the symptomless epiphytic populations with a bridge to enter the plant resulting in disease symptoms development (Carvalho et al. 2011). Previously, it has also been shown that Asiatic citrus canker was more severe when applying overhead irrigation system which also increased the incidence of this disease caused by *Xanthomonas axonopodis* pv. *citri* (Pruvost et al. 1999). In contrast, Wheeler et al. (2007) showed that overhead irrigation increased disease incidence of cotton bacterial blight by *Xanthomonas axonopodis* pv. *malvacearum* in a partially resistant cultivar; PM 2200 RR compared with drop hoses, while irrigation method did not influence disease incidence for the susceptible cultivar; PM 2326RR.

**Figure 8:**
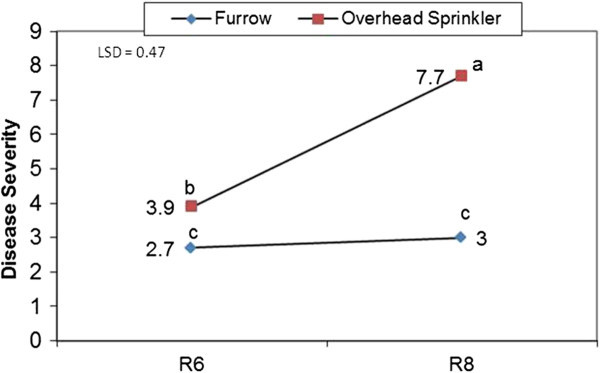
**The interaction of irrigation system and time on CBB disease severity.** With furrow irrigation, the CBB severity index did not change between the R6 (full flowering) and R8 (pod filling) bean growth stages. In contrast, CBB severity significantly increased from the R6 and R8 bean growth stages for the field using an overhead sprinkler irrigation system. The experiments were conducted in two different fields in the same research farm using a randomized complete block design with eight replicates in Arak, Iran in 2005.

Akhavan et al. (2009a) showed that the interaction of irrigation system and time was significant for disease severity. Within-furrow irrigation the CBB disease severity index had not changed between the R6 (full flowering) to R8 (pod filling) bean growth stages, but in contrast it had significantly increased during the same period in the field using an overhead sprinkler irrigation system (Figure 8).

### Epiphytic population size, disease severity and yield correlations: a brief conclusion

A significant (P<0.05) positive correlation between the epiphytic population size of *Xap* and disease severity in both the overhead irrigated field (r=0.64) and the furrow irrigated field (r= 0.44) has been reported (Akhavan et al. 2009a). The reason for the higher correlation coefficient with the overhead sprinkler irrigation system can be due to the effect of this type of irrigation on bacterial penetration to interior leaf spaces and subsequent development of disease. Similar results have been reported by other studies on the effect of windblown rain fall (Gilbertson and Maxwell 1992). Significant positive correlations between bacterial populations and disease severity have also been shown in other studies. For example, the epiphytic population of *X. campestris* pv*. vesicatoria*, the causal agent of tomato bacterial spot, was positively correlated with plant defoliation as a result of disease development (McGuire et al. 1991). Previously, Lindemann et al. (1984) also showed that the severity of brown spot of bean was correlated more consistently with epiphytic *Pseudomonas syringae* pv. *syringae* population sizes than with disease incidence. A significant (P<0.05) negative correlation between disease severity and seed yield per plant for both the furrow irrigated field (r=- 0.59) and the overhead sprinkler irrigated field (r=- 0.68) has also been reported (Akhavan et al. 2009a). Furthermore, in both systems, there was a significant (P<0.05) negative correlation between disease severity and total seed yield, but the coefficient correlation was higher under overhead sprinkler irrigation (r=- 0.83) compared to furrow irrigation (r=-0.66). This difference is likely due to more severe disease in the field under overhead sprinkler irrigation system. Since the only difference between the two fields was the type of irrigation method, it can be interpreted that the overhead sprinkler system provided several factors that encouraged epiphytic populations to increase and to establish an endophytic population of *Xap*. Overhead irrigation can provide an ideal multiplication site, i.e. a thin layer or film of water on the bean leaf surface, while also disseminating the pathogen from diseased to healthy plants. Overhead sprinkler irrigation may also help the epiphytic population of *Xap* to establish an aggressive endophytic population by facilitating the entry of bacterial cells through leaf openings followed by symptom development.

## Abbreviations

CBB: Common bacterial blight
*Xap*: *Xanthomonas axonopodis pv. phaseoli*
cfu: Colony-forming unit
